# Online E-Cigarette Information Exposure and Its Association with E-Cigarette Use among Adolescents in Shanghai, China

**DOI:** 10.3390/ijerph19063329

**Published:** 2022-03-11

**Authors:** Luojia Dai, Yaping He, Yinliang Tan, Zhiping Yu, Jingfen Zhu

**Affiliations:** 1 School of Public Health, Shanghai Jiao Tong University, Shanghai 200025, China; luojia@sjtu.edu.cn (L.D.); hypcyr@sina.com (Y.H.); melodybeata@sjtu.edu.cn (Y.T.); 2 Department of Nutrition and Dietetics, University of North Florida, Jacksonville, FL 32224-2649, USA; z.yu@unf.edu

**Keywords:** e-cigarettes, adolescents, exposure

## Abstract

E-cigarettes are widely advertised, while the potential risks of e-cigarette use have been reported among adolescents. This study assessed online e-cigarette information exposure and its association with adolescents’ e-cigarette use in Shanghai, China. A total of 12,470 students aged 13–18 years participated. A questionnaire collected information on students’ sociodemographic factors, e-cigarette information exposure, cigarette use, e-cigarette use, and e-cigarette use intention. A multivariate logistic regression was performed to assess correlates of exposure to e-cigarette information and the association between e-cigarette information exposure and e-cigarette use. Overall, 73.9% of students knew about e-cigarettes and the primary sources of information were the internet (42.4%), movies/TV (36.4%), bulletin boards in retail stores or supermarkets (34.9%), advertising flyers (33.9%), and friends (13.8%). Students who had friends using e-cigarettes were curious about e-cigarettes and showed a greater monthly allowance; smokers and females were at a higher risk of social media and website exposure. Moreover, online information exposure (social media exposure, website exposure, and total internet exposure) was significantly associated with the intention to use e-cigarettes. The enforcement of regulations on online e-cigarette content should be implemented. Moreover, efforts to prevent young people from using e-cigarettes may benefit from targeting students at a higher risk of online e-cigarette information exposure.

## 1. Introduction

The trend of tobacco purchasing and usage has gradually changed since the introduction of electronic nicotine delivery systems into the market in the mid-2000s [1]. E-cigarette use has become more and more prevalent globally among adolescents in recent years. One study showed that, in the United States, there was a considerable increase in current e-cigarette use among junior and senior high school students, from 0.6% and 1.5% in 2011 to 4.9% and 20.8% in 2018, respectively [2]. Similarly, it was also reported that, in China, although traditional cigarette smoking had decreased in adolescents, the rate of current e-cigarette use increased from 1.2% in 2014 to 2.7% in 2019 among junior high school students, and that of senior high school students had increased by a rate of 3.0% by 2019 [3]. The potentially harmful consequences of using e-cigarettes among adolescents are of great concern, which include respiratory and cardiovascular injury, nicotine addiction, neurotoxicity towards the developing brain, psychological disorders, subsequent tobacco use, etc. [4,5,6].

On the other hand, many adolescents have misconceptions or a lack of knowledge of the potential risks of e-cigarettes [7,8,9,10], which may be due to exposure to various e-cigarette commercials [11,12]. One study analyzing the content of Chinese e-cigarette manufacturer websites showed that health benefits (89%), no secondhand smoke exposure (78%), and utility for smoking cessation (67%) were mostly claimed [13], while another study compared the types and credibility of web-based information on e-cigarettes from Google (in English) and Baidu (in Chinese) and found that the Baidu websites were more likely to be owned by manufacturers and retailers, more likely to contain e-cigarette advertising, and less likely to focus on health education and providing age restriction warnings, despite the fact that the selling of e-cigarettes to minors is banned by government agencies in China [14]. Moreover, e-cigarette companies have continued to increase their advertising efforts through various channels, such as retail stores, TV/movies, newspapers/magazines, radios, and the internet [15]. Many studies have suggested that exposure to e-cigarette commercials is associated with an increased likelihood of trying e-cigarettes [11,16]. For example, studies showed that a higher exposure to e-cigarette commercials via the internet and retail stores contributed to higher odds of current e-cigarette use [17,18]. Among all marketing strategies, internet marketing has a salient role in vaping promotion, whereas retail stores are a prominent source of e-cigarette advertising [19]. 

Moreover, on the individual level, e-cigarette users may share e-cigarette-related information on internet or social media platforms, e.g., their favorite e-cigarette products, which serves to advertise e-cigarettes as well [20,21]. One review found that the discussion and promotion of e-cigarettes on social media appeared to be predominately occurring among the general public and those with vested interests; however, a noticeable silence from public health and government sectors was observed [22]. However, when adolescents came across this e-cigarette information on websites or on social media, very few questioned the validity of the content [20]. Previous research has found that both e-cigarette users and non-e-cigarette users report receiving, sharing, and searching for information on e-cigarettes on social media [23]. Since social norms are increasingly conveyed via social media, seeing e-cigarette information and use among peers on social media may suggest that using e-cigarettes is socially approved and normal [24]. Indeed, Pokharel and colleagues [25] found that e-cigarette content on social media might be linked to the increased use of e-cigarettes, and another study indicated that there were positive and significant associations between lifetime e-cigarette use and viewing peer posts as well as advertisements via social media [26]. In other studies, it was suggested that e-cigarette exposure may also lead to a greater intention to try e-cigarettes [17,27].

Although there is a consensus that exposure to e-cigarette information via the internet is positively associated with adolescents’ ever and current use, limited research has explored the influencing factors of online e-cigarette information exposure and its association with adolescents’ intentions to try e-cigarettes in the future. Despite the rapid increase in e-cigarette use among adolescents in China, the governance of e-cigarettes is still at an initial stage compared with other countries with relatively clear regulations on the use and supply of e-cigarettes [28]. China only recently incorporated the management of e-cigarettes into the “Law of the People’s Republic of China on the Protection of Minors”, in 2021 [29], but national laws on e-cigarette control have not been enacted and improvements in regulations on e-cigarette information are needed. Promisingly, the current prevalence of e-cigarette use among Chinese adolescents is relatively low compared to that of many developed countries, which is encouraging for prevention and intervention. In Shanghai, as one of the most economically developed cities in China, prevention and control among adolescents can provide a reference for other cities and regions. Therefore, this study aimed to explore exposure to e-cigarette information, especially via the internet, and its association with e-cigarette use and intention to use e-cigarettes among junior, senior, and vocational high school students in Shanghai, China.

## 2. Materials and Methods

### 2.1. Research Procedure

This study was conducted from October to December 2019 through stratified cluster random sampling. Shanghai consists of 16 districts, and all districts were initially divided into urban and suburban. One of each district was randomly selected in the first stage. A total of 20 schools (including 6 junior and 3 senior high schools, as well as 1 vocational high school from each district) were randomly selected in the second stage according to the proportion of students at school in Shanghai. Data were collected by trained investigators, and the participants were asked to fill out online questionnaires anonymously in the computer room, while teachers were asked not to be present. Of the 12,685 students who participated, 12,470 (98.3%) completed the questionnaire and were included in the analysis.

All research procedures were approved by the Shanghai Municipal Education Commission and the participating schools. All students were informed that their participation was voluntary. Written informed consent, provided before enrollment, was obtained from all students, their guardians, and school organizers, which covered the objectives, procedures, potential risks, and benefits of the study. The study was approved by the Ethics Committee of Shanghai Jiao Tong University (SJUPN-201703).

### 2.2. Measures

The questionnaire used in this study was adapted from the Global Youth Tobacco Survey (GYTS) [30], which was developed by the WHO and included the following questions.

#### 2.2.1. Sociodemographic Factors

Characteristics assessed included gender, school type, boarding situation, residence, monthly allowance, and school performance. Friends’ and parents’ e-cigarette use was classified into 2 categories: “none” and “any of them” [31].

#### 2.2.2. E-Cigarette Information Exposure

Exposure to e-cigarette information was measured by asking “Where did you get to know e-cigarettes?”: (1) flyers, (2) television or movies, (3) billboards, (e.g., in retail stores or supermarkets), (4) friends, (5) families, (6) others, and (7) never seen or heard of. Exposure to online e-cigarette information was assessed using two questions: “Have you ever seen e-cigarette advertisements on social media in the past 30 days?” and “Have you ever seen e-cigarette advertisements via websites (such as web browsing, online games, online videos, etc.) in the past 30 days?”. Response options for both were “never”, “sometimes (once or twice)”, and “often (more than 3 times)” [21]. Due to few students choosing “often”, we combined it with option “sometimes”. Thus, social media exposure and website exposure were classified into 2 categories, “no” (code 0) and “yes” (code 1), while these two variables were combined as total internet exposure. Those who chose “never” for both types of exposure were classified as no internet exposure (coded 0) and others were defined as being exposed via the internet (coded 1). 

#### 2.2.3. Cigarette Use

Cigarette use was assessed by asking whether respondents had ever tried cigarette smoking and how many days they had smoked in the past 30 days. Never smokers were defined as respondents who indicated “I never smoked even just 1 or 2 puffs”. Those respondents who reported smoking on more than one day in the past 30 days were classified as current smokers, and those who reported lifetime smoking, while not having smoked in the past 30 days, were defined as ever smokers [32].

#### 2.2.4. E-cigarette Curiosity and Use

E-cigarette curiosity was measured by asking “Are you curious about e-cigarettes?” (12). Response options were “definitely not”, “a little”, “very much”. Those who answered “definitely not” were classified as having no curiosity; all others were considered as being curious about e-cigarettes. E-cigarette use was assessed with the following items: “Have you ever tried e-cigarettes?” and “Have you ever used e-cigarettes in the past 30 days?”. The definitions of “never e-cigarette user”, “ever e-cigarette user”, and “current e-cigarette user” were the same as the cigarette-related definitions mentioned above [32]. 

#### 2.2.5. Intention to Use E-Cigarettes

The items that measured intention to use e-cigarettes were “If given the chance, would you try an e-cigarette (even just one puff)?” and “Would you try an e-cigarette if one of your best friends were to offer one to you?”. Response options were “definitely yes”, “probably yes”, “probably not”, and “definitely not” [11]. Those who reported “definitely not” to both items were categorized as “not having intention to use e-cigarettes” (coded 0) and others were classified as “having intention to use e-cigarettes” (coded 1).

### 2.3. Data Analysis 

To take the complex survey sample design into account, a weighting factor was calculated based on the selection probability of districts, the number of schools in each district, and the number of students in each school, which was then adjusted for the nonresponse. Sociodemographic factors and tobacco use characteristics associated with e-cigarette information exposure were identified by multivariate logistic regression. Adjusted odds ratios (AORs) with 95% confidence intervals (CIs) were calculated to examine the associations between exposure to e-cigarette information and ever and current e-cigarette use, as well as the intention to use e-cigarettes after controlling for covariates (such as personal characteristics, friends’ and parents’ e-cigarette use) in model 1, while adding traditional smoking status as a covariate in model 2. A *p*-value < 0.05 was considered statistically significant. All data were analyzed using SPSS 24.0 software (IBM, NY, USA).

## 3. Results

### 3.1. Descriptive Statistics

As shown in, Table 1, the total number of students was 12470, and the weighted number of students in Shanghai in 2019 was 708,765. The overall weighted sample was composed of students from junior high school (64.71%, 95%CI 63.87%–65.53%), senior high school (22.87%, 95%CI 22.14%–23.62%), and vocational high school (12.42%, 95%CI 12.01%–12.85%). The students’ mean age was 13.74 (95%CI 13.71–13.77) years old. Among all the students, slightly more student respondents were male (52.37%, 95%CI 51.43–53.32), while boarding and local students represented 14.28% and 66.29%, respectively. Nearly 20 percent of students (17.08%, 95%CI 16.41%–17.77%) reported having friends who were e-cigarette users, and less than 10 percent of them (9.75%, 95%CI 9.20%–10.33%) had at least one parent who used e-cigarettes. Additionally, 4.18% of students (95%CI 3.84%–4.55%) had ever smoked and 0.81% of them were current smokers (95%CI 0.68%–0.96%). As for e-cigarettes, approximately 20% of students (18.28%, 95%CI 17.57%–19.01%) reported being curious about e-cigarettes. Ever and current e-cigarette users accounted for 1.06% (95%CI 0.90%–1.25%) and 0.50% (95%CI 0.41%–0.62%), respectively. Moreover, the prevalence of ever (1.43%, 95%CI 1.18%–1.73%) and current (0.77%, 95%CI 0.61%–0.97%) male e-cigarette users was slightly higher than that of female students (0.66%, 95%CI 0.47%–0.92%; 0.21%, 95%CI 0.13%–0.33%).

### 3.2. Exposure to E-Cigarette Information

Nearly 74% of the students (73.9%, 95%CI 73.0%–74.7%) knew about e-cigarettes, and the top five ways for students to learn about e-cigarettes were the internet (42.4%, 95%CI 41.1%–43.3%), movies/TV (36.4%, 95%CI 35.5%–37.3%), bulletin boards in retail stores or supermarkets (34.9%, 95%CI 34.0%–35.8%), advertising flyers (33.9%, 95%CI 33.0%–34.8%), and friends (13.8%, 95%CI 13.2%–14.5%). Table 2 illustrates students’ exposure to e-cigarette information through websites or via social media during the past 30 days. Overall, 18.6% (95%CI 17.9%–19.3%) and 18.0% (95%CI 17.3%–18.7%) students had seen e-cigarette information on either websites or social media, while the total rate of exposure was 22.5% (95%CI 21.8%–23.3%).

### 3.3. Influencing Factors of Exposure to E-Cigarette Information on Websites or on Social Media

Notably, in the unadjusted model, Table 2 shows that students who were from senior and vocational high schools, boarded at school, had a greater allowance, were curious about e-cigarettes, were ever and current smokers, and were ever and current e-cigarette users were significantly associated with e-cigarette information exposure both via websites and through social media (*p* < 0.05). However, after adjusting for other factors, females were more likely than males to be exposed via social media (aOR: 1.14, 95%CI: 1.03–1.26) and via websites (aOR: 1.11, 95%CI: 1.00–1.23). Vocational high school students were more likely than junior high students to be exposed via social media (aOR: 1.16, 95%CI: 1.01–1.33). Additionally, monthly allowance was significantly associated with both means of exposure, and students who were curious about e-cigarettes were significantly more likely to be exposed on social media (aOR: 1.52, 95%CI: 1.35–1.72) and through websites (aOR: 1.51, 95%CI: 1.34–1.71). When having friends or parents using e-cigarettes, the exposure rates via these platforms were significantly higher (*p* < 0.001). Specifically, having friends using e-cigarettes was significantly associated with both exposure sources (aOR: 2.08, 95%CI: 1.85–2.34; aOR: 1.97, 95%CI: 1.75–2.23). Moreover, significant associations between smokers (ever and current) and social media exposure (aORever: 1.43, 95%CI: 1.15–1.77; aORcurrent: 2.00, 95%CI: 1.31–3.05) and website exposure (aORever: 1.35, 95%CI: 1.08–1.68; aORcurrent: 1.85, 95%CI: 1.21–2.83) were observed.

### 3.4. Association between Website or Social Media Exposure to E-Cigarette Information and E-Cigarette Use

As shown in Table 3, after controlling for gender, school type, boarding, residence, school performance, monthly allowance, friends’ e-cigarette use, and parents’ e-cigarette use in model 1, being exposed to e-cigarette information via social media was positively associated with ever e-cigarette use (aOR = 1.48, 95%CI: 1.14–1.94), current e-cigarette use (aOR = 1.62, 95%CI: 1.08–2.44), and having an intention to use e-cigarettes (aOR = 1.64, 95%CI: 1.39–1.93). Meanwhile, being exposed through websites was positively associated with ever e-cigarette use (aOR = 1.48, 95%CI: 1.13–1.94) and having an intention to use e-cigarettes (aOR = 1.46, 95%CI: 1.24–1.72). Additionally, total online exposure (social media and website exposure combined) was significantly positively associated with ever e-cigarette use (aOR = 1.37, 95%CI: 1.06–1.78) and having an intention to use e-cigarettes (aOR = 1.64, 95%CI: 1.40–1.92). However, when we added traditional smoking status as a covariate in model 2, associations were only found between intention to use e-cigarettes and social media exposure (aOR: 1.55, 95%CI: 1.31–1.84), website exposure (aOR: 1.39, 95%CI: 1.17–1.65), and total online exposure (aOR: 1.55, 95%CI: 1.32–1.83). 

## 4. Discussion

This is the first study exploring the exposure to online e-cigarette information and its association with the intention to use e-cigarettes among Chinese adolescents. The results indicated that students were highly exposed to e-cigarette information from various sources, especially through the internet. In addition, exposure to online e-cigarette information was found to be positively associated with future e-cigarette use among non-e-cigarette users.

Previous studies have shown a high rate of exposure to e-cigarette advertisement among adolescents, with the primary source of exposure being retail stores [33,34]. In the current study, the top source of exposure was the internet, followed by movies/TV, bulletin boards in retail stores or supermarkets, advertising flyers, and friends. This difference may be due to the increased regulations against offline tobacco marketing, and thus the internet has become the main forum for tobacco advertising. In this study, among all students, 42.4% of them indicated the internet as their primary exposure source. One study focused on youth exposure to vaping ads in Canada, England, and the US, indicating that the rate of ad exposure through websites or social media was approximately 40% among these countries [33]. This study found that, even in the model that adjusted for traditional smoking status, exposure to online e-cigarette information was also significantly associated with the intention to use e-cigarettes among non-e-cigarette users. This is consistent with previous research indicating that even the exposure to low-intensity e-cigarette advertising was related to the likelihood of using e-cigarettes in adolescents who had never used cigarettes or e-cigarettes before [35]. Furthermore, previous longitudinal studies found that information exposure predicted a higher likelihood of e-cigarette use later in life [36,37]. Moreover, as a new form of online marketing, advertising on social media was specifically explored in the current study. Overall, 18.6% students viewed e-cigarette information via social media through others’ posting or sharing and social media exposure was positively associated with their intention to use e-cigarettes, which is consistent with former research [35,38]. Moreover, it seemed that ad exposure was more effective at attracting non-smokers than promoting product switching in current smokers [27]. Adolescents may have been attracted because the information on social media changed their beliefs about e-cigarettes, such as the belief that e-cigarettes are safer, cleaner, have various flavors, can be used in public areas, etc. Moreover, viewing peers’ posts through social media significantly lowers adolescents’ perception of the harm caused by e-cigarettes and raises their openness and curiosity about e-cigarettes [11].

This study also identified influencing factors for students to become exposed to e-cigarette information. We did not expect that female students were more likely to be exposed, either via social media or websites, than male students. This is contrary to previous observations that male students accounted for most e-cigarette users [5,19]. However, Hébert et al. [21] reported that exposure to e-cigarette-related social media and some forms of engagement (e.g., writing, responding, and re-blogging) were more common in females. One possible explanation is that girls are significantly more likely than boys to use multiple social media platforms [39], which increases their potential to access to more e-cigarette information. Additionally, although the prevalence of girls’ ever and current e-cigarette use (0.7%, 0.2%) was still lower than those of boys (1.4%, 0.8%), the values were higher than those of Chinese female adults (0.5%, 0.1%) [40]. Additionally, it was found by Moore et al. [41] that the gender difference in e-cigarette use was less significant than that in traditional smoking. Moreover, students from a vocational high school were more likely to be exposed to e-cigarette information via social media, which may be related to their lower academic pressure, less strict school management, and wider social interactions. Therefore, more attention should be paid to protecting teenage girls and vocational high school students from using e-cigarettes.

Another significant influencing factor on e-cigarette information exposure was a high monthly allowance. Earlier research found that teenagers with a higher monthly allowance were more likely to use e-cigarettes [42]. This may be explained by the notion that, with more money at their disposal, they were less affected by the price of e-cigarettes, as previous studies discovered that higher e-cigarette prices appeared to be associated with reduced e-cigarette use [43,44], shedding light on the possibility of raising e-cigarette prices, such as via taxes, to reduce adolescents’ e-cigarette exposure and usage. Moreover, reducing the amount of monthly allowance may be a new method of future intervention. In addition, it may not be surprising that students who smoked and were curious about e-cigarettes were more likely to view e-cigarette information, which is consistent with past research [33,45], because they were found to have significantly more favorable attitudes towards e-cigarettes [7].

Previously, it was found that a social environment favorable to e-cigarettes (e.g., friends’ use of and positive attitudes toward the use of e-cigarettes) was associated with a greater likelihood of cigarette use [46]. Sawdey et al. [26] also indicated that current e-cigarette use was significantly associated with viewing peer posts on social media, and significant main effects of peer-generated posts on willingness and intention to use e-cigarettes, positive attitudes, and greater norm perceptions among adolescents were found [24]. These findings support the theory that one’s behavior is greatly influenced by one’s peers, social network, and social environment [47]. In our study, friends were not the primary source of e-cigarette knowledge for adolescents. However, having friends using e-cigarettes was significantly associated with exposure to e-cigarette information via websites or on social media. A possible explanation is that, since social media has become an inseparable part of adolescents’ daily lives, the influence of online social networks through social media is independent of the influence of in-person social networks [38]. Future interventions can utilize online social networks as media platforms to appropriately disseminate e-cigarette information and reduce e-cigarette use among young people. 

It is important to acknowledge that there are some limitations to this study. Due to the nature of self-reported data, the responses may have over- or underestimated teenagers’ e-cigarette exposure because of recall bias, and the rate of students’ e-cigarette use and smoking might be underestimated as some of the students may not have reported it for fear of violating school rules against smoking. In addition, this was a cross-sectional study; therefore, causal relationships between the variables cannot be determined. This study only focused on whether students were exposed to e-cigarette information; more factors could be explored in future studies—for example, the measurement of media use (recall of time, time-use diaries, type of social media platforms used, level of media use, etc.) and evaluation of the content of electronic cigarette information (e.g., for or in support of vaping versus against and in support of regulation) [24]. Moreover, the data from this study, which are based on adolescents in Shanghai, are not representative of China as a whole and are only a microcosm of e-cigarette use and exposure among urban Chinese adolescents. Regardless, Shanghai is a mega-city, with the greatest and fastest economic development in China. Students in Shanghai are more likely to be exposed to new and fashionable products such as e-cigarettes, so their prevention and control is very important, and this information can be used as a reference for other cities and regions. Moreover, the finding that exposure to e-cigarette information via websites or on social media was linked to teenagers’ intentions to use e-cigarettes is novel and calls for longitudinal studies to further assess the impact of e-cigarette exposure on teenagers’ e-cigarette initiation. 

## 5. Conclusions

This study reported a high exposure rate of e-cigarette information among adolescents in Shanghai, China, and it was found that the main avenue for their exposure was through the internet. It was also found that exposure to e-cigarette information via websites or on social media may increase the risk of e-cigarette use intention in this population. The enforcement of regulations on online e-cigarette information should be implemented. Moreover, our study suggested that efforts to prevent young people from using e-cigarettes may benefit from targeting students who have friends using e-cigarettes, are curious about e-cigarettes, have a higher monthly allowance, and are smokers (ever and current) and females, as these groups are at higher risk of being exposed to e-cigarette information.

## Figures and Tables

**Table 1:** Baseline characteristics (N = 12,470).

		Weighted		Unweighted
	%	95%CI	Number	N (%)
Age (mean, 95%CI)				
	13.74	13.71–13.77	708,765	14.45 (14.42–14.49)
Gender				
Male	52.37	51.43–53.32	371,209	6736 (54.02)
Female	47.63	46.68–48.57	337,556	5734 (45.98)
Type of school				
Junior high school	64.71	63.87–65.53	458,613	5842 (46.85)
Senior high school	22.87	22.14–23.62	162,091	3032 (24.31)
Vocational high school	12.42	12.01–12.85	88,062	3596 (28.84)
Boarding in school				
Yes	14.28	13.73–14.85	101,211	2821 (22.62)
No	85.72	85.15–86.27	607,554	9649 (77.38)
Residence				
Local	66.29	65.38–67.19	469,857	8350 (66.96)
Non-local	33.71	32.81–34.62	238,909	4120 (33.04)
Monthly allowance				
<200 RMB (<31.5 USD)	34.64	33.74–35.55	245,506	4206 (33.73)
200–599RMB (31.5-94.5USD)	41.37	40.44–42.30	293,192	5204 (41.73)
≥600 RMB (≥94.5 USD)	23.99	23.20–24.81	170,068	3060 (24.54)
School performance				
Top 25%	61.19	60.29–62.09	433,705	6537 (52.42)
Average	26.47	25.67–27.28	187,603	3830 (30.71)
Bottom 25%	12.34	11.79–12.92	87,458	2103 (16.86)
Friends’ e-cigarette use				
No	82.92	82.23–83.59	587,709	9908 (79.45)
Yes	17.08	16.41–17.77	121,057	2562 (20.55)
Parents’ e-cigarette use				
No	90.25	89.67–90.80	639,690	11,271 (90.38)
Yes	9.75	9.20–10.33	69,075	1199 (9.62)
E-cigarette curiosity				
No	81.72	80.99–82.43	579,207	10,020 (80.40)
Yes	18.28	17.57–19.01	129,559	2450 (19.60)
Traditional smoking status				
Never	95.01	94.62–95.37	673,394	11,663 (93.53)
Ever	4.18	3.84–4.55	29,635	644 (5.16)
Current	0.81	0.68–0.96	5746	163 (1.31)
E-cigarette use				
Never	98.44	98.22–98.63	697,680	12,184 (97.71)
Ever	1.06	0.90–1.25	7522	175 (1.40)
Current	0.50	0.41–0.62	3564	111 (0.89)

**Table 2:** Online e-cigarette information exposure and its influencing factors (N = 708,765).

	Social Media Exposure					Website Exposure				
	%(95%CI)	cOR(95%CI) ^a,b^	*p*	aOR(95%CI) ^a,c^	*p*	%(95%CI)	cOR(95%CI) ^a,b^	*p*	aOR(95%CI) ^a,c^	*p*
Gender										
Male	18.3 (17.3–19.2)	Ref = 1		Ref = 1		17.9 (17.0–18.9)	Ref = 1		Ref = 1	
Female	18.9 (17.9–19.9)	1.04 (0.94–1.14)	0.450	1.14 (1.03–1.26)	0.012	18.2 (17.2–19.2)	1.02 (0.93–1.13)	0.670	1.11 (1.00–1.23)	0.047
School type										
Junior high school	16.8 (15.9–17.8)	Ref = 1		Ref = 1		16.2 (15.3–17.2)	Ref = 1		Ref = 1	
Senior high school	18.8 (17.5–20.3)	1.15 (1.02–1.28)	0.019	0.92 (0.81–1.05)	0.229	19.0 (17.7–20.5)	1.21 (1.08–1.36)	0.001	0.97 (0.85–1.11)	0.691
Vocational high school	24.6 (23.4–25.8)	1.61 (1.46–1.77)	<0.001	1.16 (1.01–1.33)	0.031	23.4 (22.2–24.6)	1.58 (1.43–1.73)	<0.001	1.10 (0.96–1.27)	0.165
Boarding in school										
No	18.1 (17.3–18.8)	Ref = 1		Ref = 1		17.4 (16.7–18.2)	Ref = 1		Ref = 1	
Yes	21.3 (19.9–22.9)	1.23 (1.11–1.37)	<0.001	0.92 (0.80–1.05)	0.214	21.6 (20.1–23.1)	1.30 (1.17–1.45)	<0.001	1.00 (0.87–1.15)	0.979
Residence										
Local	18.7 (17.9–19.6)	Ref = 1		Ref = 1		18.2 (17.3–19.0)	Ref = 1		Ref = 1	
Non-local	18.2 (17.0–19.4)	0.96 (0.87–1.06)	0.460	0.97 (0.87–1.08)	0.560	17.7 (16.6–19.0)	0.97 (0.88–1.07)	0.568	0.98 (0.87–1.09)	0.670
Monthly allowance										
<200 RMB (<31.5 USD)	15.9 (15.0–16.8)	Ref = 1		Ref = 1		15.4 (14.6–16.3)	Ref = 1		Ref = 1	
200–599 RMB (31.5–94.5 USD)	21.2 (19.9–22.6)	1.42 (1.28–1.58)	<0.001	1.29 (1.14–1.45)	<0.001	20.5 (19.2–21.8)	1.41 (1.27–1.57)	<0.001	1.28 (1.13–1.44)	<0.001
≥600 RMB (≥94.5 USD)	25.1 (23.2–27.0)	1.77 (1.57–2.00)	<0.001	1.44 (1.23–1.67)	<0.001	24.6 (22.7–26.5)	1.78 (1.58–2.02)	<0.001	1.45 (1.24–1.68)	<0.001
School performance										
Top 25%	17.3 (16.1–18.5)	Ref = 1		Ref = 1		17.5 (16.3–18.7)	Ref = 1		Ref = 1	
Average	19.1 (18.0–20.2)	1.13 (1.01–1.26)	0.026	1.11 (0.99–1.24)	0.082	18.1 (17.1–19.2)	1.05 (0.94–1.17)	0.404	1.02 (0.91–1.14)	0.740
Bottom 25%	19.5 (18.1–21.0)	1.16 (1.03–1.31)	0.015	1.08 (0.95–1.23)	0.251	18.7 (17.3–20.1)	1.08 (0.96–1.23)	0.196	1.00 (0.88–1.14)	0.981
Friends’ e-cigarette use										
No	15.7 (15.0–16.4)	Ref = 1		Ref = 1		15.3 (14.6–16.1)	Ref = 1		Ref = 1	
Yes	31.4 (29.6–33.3)	2.46 (2.23–2.73)	<0.001	2.08 (1.85–2.34)	<0.001	30.1 (28.4–32.0)	2.38 (2.15–2.64)	<0.001	1.97 (1.75–2.23)	<0.001
Parents’ e-cigarette use										
No	18.1 (17.3–18.8)	Ref = 1		Ref = 1		17.5 (16.8–18.2)	Ref = 1		Ref = 1	
Yes	23.1 (20.8–25.7)	1.37 (1.18–1.58)	<0.001	1.09 (0.93–1.28)	0.307	23.0 (20.6–25.5)	1.41 (1.21–1.63)	<0.001	1.15 (0.98–1.35)	0.077
E-cigarette curiosity										
No	16.6 (15.8–17.3)	Ref = 1		Ref = 1		16.1 (15.4–16.8)	Ref = 1		Ref = 1	
Yes	27.3 (25.5–29.1)	1.89 (1.70–2.10)	<0.001	1.52 (1.35–1.72)	<0.001	26.5 (24.7–28.3)	1.88 (1.69–2.09)	<0.001	1.51 (1.34–1.71)	<0.001
Traditional smoking status										
Never	17.6 (16.9–18.4)	Ref = 1		Ref = 1		17.2 (16.2–17.9)	Ref = 1		Ref = 1	
Ever	28.5 (24.8–32.5)	1.87 (1.54–2.27)	<0.001	1.43 (1.15–1.77)	<0.001	27.0 (23.4–30.9)	1.78 (1.46–2.17)	<0.001	1.35 (1.08–1.68)	0.005
Current	44.9 (36.4–53.6)	3.80 (2.67–5.43)	<0.001	2.00 (1.31–3.05)	<0.001	42.2 (33.9–51.1)	3.53 (2.46–5.06)	<0.001	1.85 (1.21–2.83)	0.008
E-cigarette use										
Never	18.0 (17.3–18.8)	Ref = 1		Ref = 1			Ref = 1		Ref = 1	
Ever	31.9 (24.6–40.2)	2.13 (1.47–3.07)	<0.001	0.85 (0.56–1.29)	0.458	2.28 (1.58–3.27)	2.28 (1.58–3.27)	<0.001	0.97 (0.64–1.48)	0.904
Current	42.7 (32.9–53.1)	3.38 (2.22–5.16)	<0.001	0.98 (0.60–1.58)	0.923	3.01 (1.96–4.61)	3.01 (1.96–4.61)	<0.001	0.92 (0.57–1.51)	0.754
Total	18.6 (17.9–19.3)					18.0 (17.3–18.7)				

^a^ compared with no exposure; ^b^ cOR stands for crude odds ratio; ^c^ model adjusted for gender, school type, boarding in school, residence, school performance, monthly allowance, friends’ e-cigarette use, parents’ e-cigarette use, e-cigarette curiosity, traditional smoking status, and e-cigarette use.

**Table 3:** Online e-cigarette information exposure and e-cigarette use risk among adolescents.

	Ever E-Cigarette Use ^c^				Current E-Cigarette Use ^c^				E-Cigarette Use Intention ^d^			
	Model 1		Model 2		Model 1		Model 2		Model 1		Model 2	
	aOR(95%CI) ^a^	*p*	aOR(95%CI) ^b^	*p*	aOR(95%CI) ^a^	*p*	aOR(95%CI) ^b^	*p*	aOR(95%CI) ^a^	*p*	aOR(95%CI) ^b^	*p*
Social media exposure												
No	Ref = 1		Ref = 1		Ref = 1		Ref = 1		Ref = 1		Ref = 1	
Yes	1.48 (1.14–1.94)	0.001	1.14 (0.81–1.61)	0.443	1.62 (1.08–2.44)	0.019	1.40 (0.88–2.24)	0.156	1.64 (1.39–1.93)	<0.001	1.55 (1.31–1.84)	<0.001
Websites exposure												
No	Ref = 1		Ref = 1		Ref = 1		Ref = 1		Ref = 1		Ref = 1	
Yes	1.48 (1.13–1.94)	0.004	1.24 (0.88–1.75)	0.223	1.50 (0.99–2.27)	0.054	1.26 (0.77–2.04)	0.354	1.46 (1.24–1.72)	<0.001	1.39 (1.17–1.65)	<0.001
Total internet exposure												
No	Ref = 1		Ref = 1		Ref = 1		Ref = 1		Ref = 1		Ref = 1	
Yes	1.37 (1.06–1.78)	0.017	1.05 (0.75–1.47)	0.769	1.43 (0.96–2.14)	0.076	1.19 (0.75–1.89)	0.467	1.64 (1.40–1.92)	<0.001	1.55 (1.32–1.83)	<0.001

^a^ Model adjusted for gender, school type, boarding, residence, school performance, monthly allowance, friends’ e-cigarette use, and parents’ e-cigarette use. ^b^ Model adjusted for gender, school type, boarding, residence, school performance, monthly allowance, friends’ e-cigarette use, parents’ e-cigarette use, and traditional smoking status. ^c^ among all participants (N = 708,765). ^d^ among non-e-cigarette users (N = 697,680).

## Data Availability

The data presented in this study are available on request from the corresponding author. The data are not publicly available due to the ethical facts that adolescents are a vulnerable group and their privacy should be protected.
